# Intramuscular Connective Tissue Differences in Spastic and Control Muscle: A Mechanical and Histological Study

**DOI:** 10.1371/journal.pone.0101038

**Published:** 2014-06-30

**Authors:** Marije de Bruin, Mark J. Smeulders, Michiel Kreulen, Peter A. Huijing, Richard T Jaspers

**Affiliations:** 1 Department of Plastic, Reconstructive and Hand Surgery, Academic Medical Center, Amsterdam, The Netherlands; 2 Department of Plastic, Reconstructive and Hand Surgery, Red Cross Hospital, Beverwijk, The Netherlands; 3 Laboratory for Myology, MOVE Research Institute Amsterdam, Faculty of Human Movement Sciences, VU University Amsterdam, Amsterdam, The Netherlands; Stem Cell Research Institute, Belgium

## Abstract

Cerebral palsy (CP) of the spastic type is a neurological disorder characterized by a velocity-dependent increase in tonic stretch reflexes with exaggerated tendon jerks. Secondary to the spasticity, muscle adaptation is presumed to contribute to limitations in the passive range of joint motion. However, the mechanisms underlying these limitations are unknown. Using biopsies, we compared mechanical as well as histological properties of flexor carpi ulnaris muscle (FCU) from CP patients (n = 29) and healthy controls (n = 10). The sarcomere slack length (mean 2.5 µm, SEM 0.05) and slope of the normalized sarcomere length-tension characteristics of spastic fascicle segments and single myofibre segments were not different from those of control muscle. Fibre type distribution also showed no significant differences. Fibre size was significantly smaller (1933 µm^2^, SEM 190) in spastic muscle than in controls (2572 µm^2^, SEM 322). However, our statistical analyses indicate that the latter difference is likely to be explained by age, rather than by the affliction. Quantities of endomysial and perimysial networks within biopsies of control and spastic muscle were unchanged with one exception: a significant thickening of the tertiary perimysium (3-fold), i.e. the connective tissue reinforcement of neurovascular tissues penetrating the muscle. Note that this thickening in tertiary perimysium was shown in the majority of CP patients, however a small number of patients (n = 4 out of 23) did not have this feature. These results are taken as indications that enhanced myofascial loads on FCU is one among several factors contributing in a major way to the aetiology of limitation of movement at the wrist in CP and the characteristic wrist position of such patients.

## Introduction

Spasticity in the forearm due to cerebral palsy (CP) is associated with a limited range of active and passive movement around the wrist and elbow. The flexor carpi ulnaris muscle (FCU) is held largely responsible for the limited range of motion and the contracture around the wrist. Presumed muscle adaptation induced by longstanding spasticity is regarded as the major contributor to the passive movement limitation. Therefore, this muscle is frequently subject of surgical treatment of the spastic arm [1]. In patients with CP, development of lower extremity muscles (triceps surae and hamstrings) has been reported to be compromised, causing shortness [2], [3] and/or an increased passive muscle stiffness [4], [5], [6].

The mechanisms by which spasticity of the FCU results in a limited passive movement around the wrist and elbow are unknown. Several pathophysiological mechanisms may underlie the altered spastic FCU development. Due to the spasticity and the related reduced ability of CP patients to extend the wrist, FCU is largely maintained in a shortened position. Based on effects found for immobilization of experimental animal muscle in a shortened position [7], [8], both impeded growth of myofibre diameter and diminished addition of serial sarcomeres within myofibres have been presumed in spastic muscle [9]. However, to our knowledge, quantitative data regarding spasticity related differences in serial sarcomere number are insufficient and hard to obtain, as this requires isolation of myofibres along their full length.

For pennate muscle, such as FCU, myofibre diameter is also a major determinant of both muscle slack and optimum length [10], [11]. As such, changes in myofibre cross-sectional size could result in a shift in the muscle operating length range *in vivo*, and affect the wrist range of motion. Regarding the cross-sectional size of spastic myofibres, both atrophy and hypertrophy of slow, as well as fast myofibre types, have been reported in muscles from different limbs without a clear relation to the degree of limitation of joint movement [12], [13], [14], [15], [16], [17]. In addition, some studies reported similar cross-sectional areas of spastic and control myofibres comparing several muscles from different limbs [18], [19]. From the above we can conclude that alleged muscle stiffness is not unequivocally related to myofibre cross-sectional size and muscle shortness in CP.

Other factors that may affect muscle stiffness are (1) a change in the intrinsic, mechanical properties of the myofibres (i.e. myofibre stiffness [20]), (2) the intramuscular connective tissue [13], [21], or (3) altered myofascial loads of the epimuscular myofascial connections of the spastic muscle with extramuscular connective tissues, synergists and/or antagonist muscles [22].

Single myofibre segments obtained from different spastic muscles of the forearm have been reported to be stiffer than those of control muscle [20]. However, fascicle segments of spastic muscles have been reported to be more compliant than similar segments in control muscle, suggesting spasticity related deterioration of intramuscular connective tissue [23]. Furthermore, the analysis of the amount of connective tissue in human muscle tissue obtained from muscles in the leg and arm has shown diverse results (cf. [6], [13], [16], [18], [21], [23]). Above-mentioned variability in results may exist because comparisons were made between biopsies obtained from different muscles within one limb, muscles of different limbs or from biopsies taken from different locations within a muscle. The purpose of this study was to test the hypothesis that the limited range of wrist motion is caused by enhanced stiffness of spastic muscle as affected by intrinsic characteristics of myofibres and fascicles. To test this, we investigated mechanical and histological characteristics of spastic and healthy muscle biopsies taken from the same part of FCU muscle.

## Methods

### Ethics

All subjects older than 18 years of age gave written informed consent for the study. Subjects younger than 18 years of age participated in this study with written informed consent from their parents (as well as from the children themselves, if aged 12 years or over). The study and informed consent procedure were approved by the local Medical Ethics Committee of the Academic Medical Centre of Amsterdam. The study adhered to the ethical guidelines of the 1975 Declaration of Helsinki

### Subjects

Undergoing upper extremity surgery between 2006 and 2009, 29 patients (mean age 19 years, range 5–40, 15 male) with CP and a Zancolli type IIa or IIb grasp and release pattern [24] took part in the study. Patients that have a type Zancolli IIa or IIb grasp and release pattern, have active finger extension that is accompanied by a wrist flexion angle greater than 20°. Furthermore, in type Zancolli IIa pattern the wrist can be actively extended with flexed fingers whereas in type Zancolli IIb pattern there is no active wrist extension [24].

Healthy control subjects (n = 10; mean age 45 years; range 21–62; 3 male), who required upper extremity surgery due to cut or ruptured tendons (n = 5), bone deformities (n = 3), or traumas (n = 2) were also studied. Because of technical failure causing loss of the frozen parts of the biopsies, one of the control subjects and three to six of the patients were excluded from the histological measurements (depending on the type of staining).

### Collection and storage of muscle biopsies

During surgery, muscle biopsies (size ∼10×0.5×5 mm) were collected from the most distal part of FCU. Each biopsy was divided in two parts. The long axis of both samples was taken parallel to the longitudinal direction of the myofibres. One part to be used for mechanical measurements was put in a 50% muscle relaxing, 50% glycerol solution and stored at −20°C until further use. The relaxing solution consisted of: EGTA, 7.5 mM; potassium proprionate, 170 mM; magnesium acetate, 2 mM; imidazole, 5 mM; creatine phosphate, 10 mM; adenosine triphosphate (ATP), 4 mM; leupeptin, 17 µg/ml; and protease inhibitor E64, 4 µg/ml. Storage time did not affect sarcomere length-tension strain characteristics. All chemicals were obtained from Sigma Aldrich (the Netherlands) unless stated otherwise. The other part, to be used for histological and histochemical analysis, was frozen and stored in liquid nitrogen for maximally one week. Using a cryomicrotome, serial cross-sections (10 µm thick) were cut at −25°C, and then stored at −80°C until further use.

### Mechanical measurements

Segments of a fascicle (containing 15–30 myofibres, mean length 6.0 mm ±1.2), as well as single myofibre segments (mean length 5.6 mm ±1.7) were microscopically dissected in 12 of the 29 patient samples and all control samples. Force transducer limitations (maximal load 0.12 N) limited the size of fascicle segments that could be measured. Small platinum hooks (50 µm thick) were tied to both ends of the segments using 20 µm diameter polyamide thread (Ethicon).

At a mean sarcomere length of 2.7 µm, the largest (a) and smallest (b) segment diameters were measured at three locations along the length of the segment (in the middle and 1 mm from each endpoint) by rotating the segment and using an ocular scale. The cross-sectional area at each of the three positions along the segment was calculated assuming an ellipsoidal cross-section (1/4*π times the product of the largest and smallest segment diameters). The mean of these three values was taken as the cross-sectional area of the segment.

**Figure 1:**
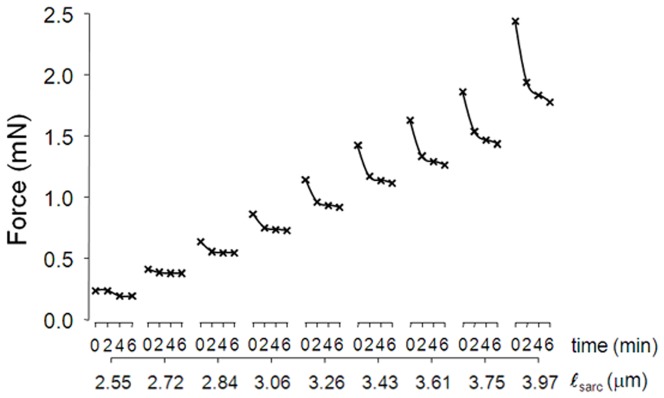
Stress relaxation of myofibres and fascicles at different sarcomere lengths. Representative passive forces of a spastic single myofibre segment from a spastic FCU as a function of sarcomere length (ℓsarc). Forces were measured immediately (time  = 0 min), and 2, 4, and 6 minutes after imposing each sarcomere strain increment. Note that effects of stress relaxation are small after at least 4 minutes, therefore sarcomere length-tension curves of myofibre and fascicle segments were assessed based on the forces measured after 4 minutes.

One end of the segment was connected to a force transducer (AE801, SensoNor, Horten, Norway) and the other end was connected to a micromanipulator. To measure passive elastic properties, the myofibre and fascicle segments were elongated in steps of 250 µm, starting at passive slack length. Measurements were performed in 100% muscle-relaxing solution described in paragraph “Collection and storage of muscle biopsies” (20°C). In a pilot study, tensions and sarcomere lengths were measured immediately following lengthening and after 2, 4 and 6 minutes, to take the effects of stress-relaxation into account (Fig. 1). Such effects were present up to 4 minutes. Therefore, all further analyses were carried out on tensions measured 4 minutes after imposing each strain increment. The segments were elongated until sarcomere lengths were beyond the physiological range (≥4.1 µm) [25], [26] or until mechanical failure occurred.

To compare mechanical properties of the myofibre and fascicle segments, tensions were normalized to the cross-sectional area. Data for tension as a function of sarcomere length were least square fitted by a polynomial function. The polynomial order that best described the experimental data was selected with one-way analysis of variance (ANOVA). Third order polynomial curves turned out to provide the best description for all curves (mean R^2^_myofibre_ = 0.993±0.005; mean R^2^_fascicle_ = 0.990±0.01). These polynomials were used for averaging of data and calculation of standard errors at set sarcomere lengths. The slope of the fitted curve (i.e. stiffness) was calculated within the physiological range by differentiation and considered an estimate of static passive stiffness of the myofibres and fascicles.

### Histology

#### Hematoxilin and eosine (HE) staining

To compare global morphology and spatial distribution of myonuclei, sections were fixed for 10 min in 4% formaldehyde in 0.1 M sodium phosphate buffer, pH 7.4, and stained with hematoxylin and eosin [27].

#### Myofibrillar ATPase staining

Myofibre typing was performed according to Brook and Kaiser [28]. Optimal myofibre type differentiation was attained at pH 4.5, at which type I myofibres stained black, type IIA myofibres white and fibres that express type IIX or co-express type A and X stained (dark) grey (referred to as IIAX) [29] .

#### Sirius red staining

Connective tissue was visualized using a modification of the Sirius red staining protocol by Junquiera et al. [30]. To minimize cytoplasmic staining, sections were first fixed in acetone at 0°C for 30 minutes and subsequently in Bouin's solution (75 ml Picric acid, 25 ml 10% formalin, and 5 ml glacial acetic acid) at 20°C for 30 minutes. Following this, sections were stained using a picrosirius red F3BA 0.1% (C.I. 35782; Direct red 80; Sigma Aldrich, the Netherlands) for 30 minutes in a dark environment. After staining, sections were washed in 10 mM HCl and then rinsed two times in absolute ethanol. Subsequently, the sections were submerged in Xylene for 10 seconds and again in Xylene for 2 minutes. Finally, slides were covered with Entallan mounting medium (Merck, Darmstadt, Germany) and a glass cover slip.

#### Image analyses

Images were obtained using a Zeiss microscope (Axioskop 50) coupled to a AxioCam (Zeiss, Germany) and were analysed with ImageJ (v. 1.41o,; USA National Institute of Health, http://rsbweb.nih.gov/ij/). Within each ATPase-stained sample cross-section, the cross-sectional area of the myofibres (A_MF_) and fibre perimeter were measured in at least 30 randomly selected cells per myofibre type by manually tracking the fibres. Sections with myofibres at the edge of sections and obliquely cut fibres (circularity <0.30) were excluded from analysis. To measure connective tissue parameters in Sirius red stained sections, contrast between yellow cytoplasm and red connective tissue was enhanced by image-filtering (green-filter [31]) using ImageJ. Subsequently, a binary threshold was applied such that a black overlay covered only the gray values above threshold (i.e. the connective tissue (red stained) areas). For all measurements, we used the Maximum Entropy threshold method by Jarek Sasha (http://ij-plugins.sf.net) prepared for ImageJ. This method minimizes erroneous detection of collagen in the cytoplasm.

Within the fascicle, the endomysium is defined as the connective tissue surrounding single myofibres. In the images, we selected areas containing only myofibres and endomysium. The black areas within the selection represented the absolute endomysium cross-sectional area (A_E_). To normalize for myofibre cross-sectional area (A_MF_), the absolute surface area of endomysium per myofibre (A_E_/MF) was determined as: A_E_ divided by the number of myofibres in the selected regions containing only endomysia and myofibres. The number of myofibres in this region was estimated by dividing the total surface area of the myofibres by the mean A_MF_ of the myofibres (measured on the myofibres of which the entire cross-section was contained within the selected region). Mean endomysium thickness per myofibre (ℓ_E_) was calculated by dividing A_E_/MF by the measured mean perimeter of the myofibres fulfilling the above criterion.

Traditionally, two domains are distinguished for the perimysium: primary perimysium embedding smallest fascicles of myofibres and secondary perimysium embedding larger fascicles, containing several primary fascicles [32]. We propose distinction of a specialized third level of perimysium. This tertiary perimysium borders parts of the secondary fascicles, but is thickened compared to secondary perimysium and traverses the muscle. The thickness of primary and secondary perimysium in cross-sections (ℓ_P1&P2_) was measured every 25 µm along the perimeter of the fascicle of at least 5 fascicles within the cross-section. The tertiary perimysium thickness (ℓ_P3_) was measured every 25 µm along its length over a length of at least 1 mm.

### Assessment of functional implication of extramuscular connections

To visualize effects extramuscular myofascial connections on length changes of FCU *in vivo*, we collected video data of a boy (14 years old) undergoing tendon transfer surgery. The first step before transfer of the FCU tendon was tenotomy performed just proximal to the pisiform bone causing complete release from its insertion but leaving extramuscular connections of the fascial surroundings to the muscle belly intact. After FCU distal tenotomy the wrist was moved towards maximal flexion and extension by the surgeon. The distal tendon of the FCU and its surroundings was filmed while moving the wrist dynamically. In a subsequent step, FCU muscle belly was dissected partially free from its surrounded connection tissue and photographs were taken.

### Statistics

A mixed design repeated measures analysis of covariance (ANCOVA) with one between subjects factor (SPSS Statistics 17.0) and age as covariate was performed to determine if there was a difference between the control and spastic passive sarcomere length-tension curves and the slopes of these curves of myofibre segments and fascicle segments. Slack lengths, muscle morphology and histochemical results of control and spastic groups were compared using *t*-tests. A non-parametric test of independent samples was used to compare myofibre type distributions and perimysial tissue variables, as these were not normally distributed. Non-parametric Spearman's correlations were used to test for age effects (A_MF_, fibre type proportions, and several connective tissue variables). As the CP group consisted of both children and adults, and literature shows that myofibre size in typically developing children generally increases up to the age of 20 years (cf. [33], [34], [35], [36], [37]), additional *t*-tests were performed to test for differences in A_MF_ between CP and control subjects both aged ≥20 years (CP n = 7; controls n = 10). For the CP group, effects of sex were tested using *t*-tests. Differences were considered significant at *p*<0.05. All data are presented as mean±standard error of the mean (SEM).

## Results

### Subject characteristics

As a result of an age restriction for control subjects due to medical ethical considerations, control subjects (*n* = 10, mean age 44.8±4.3 years) were significantly older than spastic patients (*n* = 29, mean age 18.9±1.6 years).

### Mechanical characteristics

**Figure 2:**
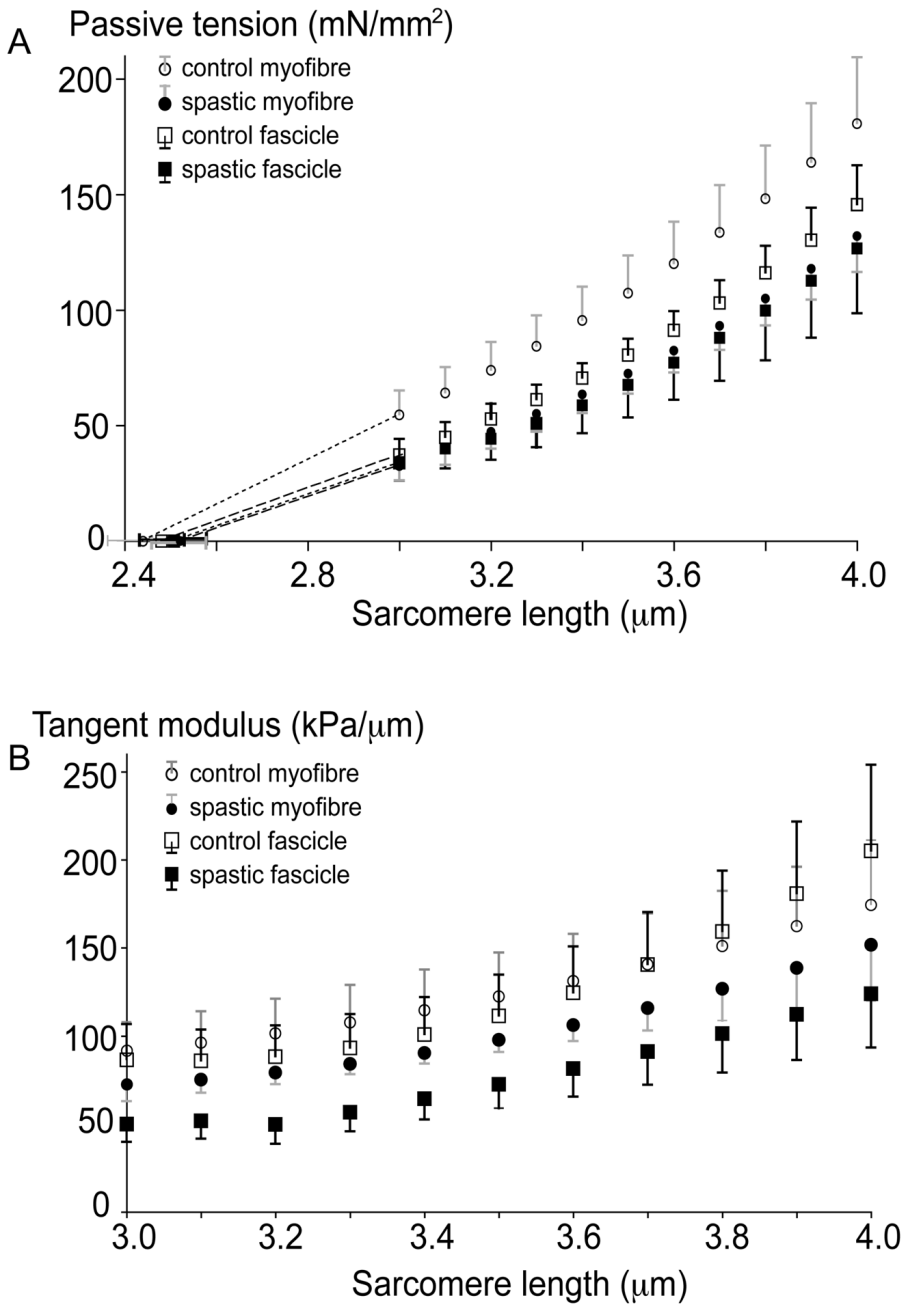
Passive length-tension characteristics of single myofibre segments and fascicle segments in spastic and control FCU. (**A**) passive tension as function of sarcomere length. Sarcomere length-passive tension curves were neither significantly different comparing spastic and control (n = 10) single myofibre segments (both n = 10), nor comparing spastic and control fascicle segments (both n = 10). The same was found for comparing single myofibre segments to fascicle segments within each group. (**B**) slopes of passive length-tension as function of sarcomere length. The curves describing the slopes were neither significantly different comparing spastic and control (n = 10) single myofibre segments (both n = 10), nor comparing spastic and control fascicle segments (both n = 10). The same was found for comparing single myofibre segments with fascicle segments within each group. Means and SEM are plotted.

All myofibre segments could be strained up to a sarcomere length of at least 4.0 µm. However, for six out of 22 fascicle segments, force transducer limitations prevented us from determining stress-strain characteristics up to this length (two were strained up to 3.5 and 3.6 respectively, and four others were strained up to 3.8 µm). Mean sarcomere slack length was neither significantly different between CP (2.52±0.08 µm) and control myofibre segments (2.44±0.06 µm) nor between CP (2.51±0.07 µm) and control fascicle segments (2.49±0.05 µm) (Fig. 2A). Over the whole range of sarcomere lengths, passive tensions of spastic and control myofibre segments and fascicle segments did not differ significantly (Fig. 2A). No significant differences were found between the passive length-tension curves of control and spastic myofibre segments or between control and spastic fascicle segments. No interaction was present with main factors sarcomere length and CP and type of segment (myofibre or fascicle).

Similarly, sarcomere length-slope of tension curves of spastic myofibre segments and fascicle segments did not differ from sarcomere length-slope of tension curves of control myofibre segments and fascicle segments (Fig. 2B). Again, no interactions were found between sarcomere length, spasticity and type of muscle segment (fibre or fascicle). Thus, increasing sarcomere length had the same result on slope of both control and spastic myofibre segments and fascicle segments.

For both myofibres and fascicle segments, mechanical characteristics were not related to age nor was there a difference between male and female CP patients.

### Myofibre histology

#### HE staining

**Figure 3:**
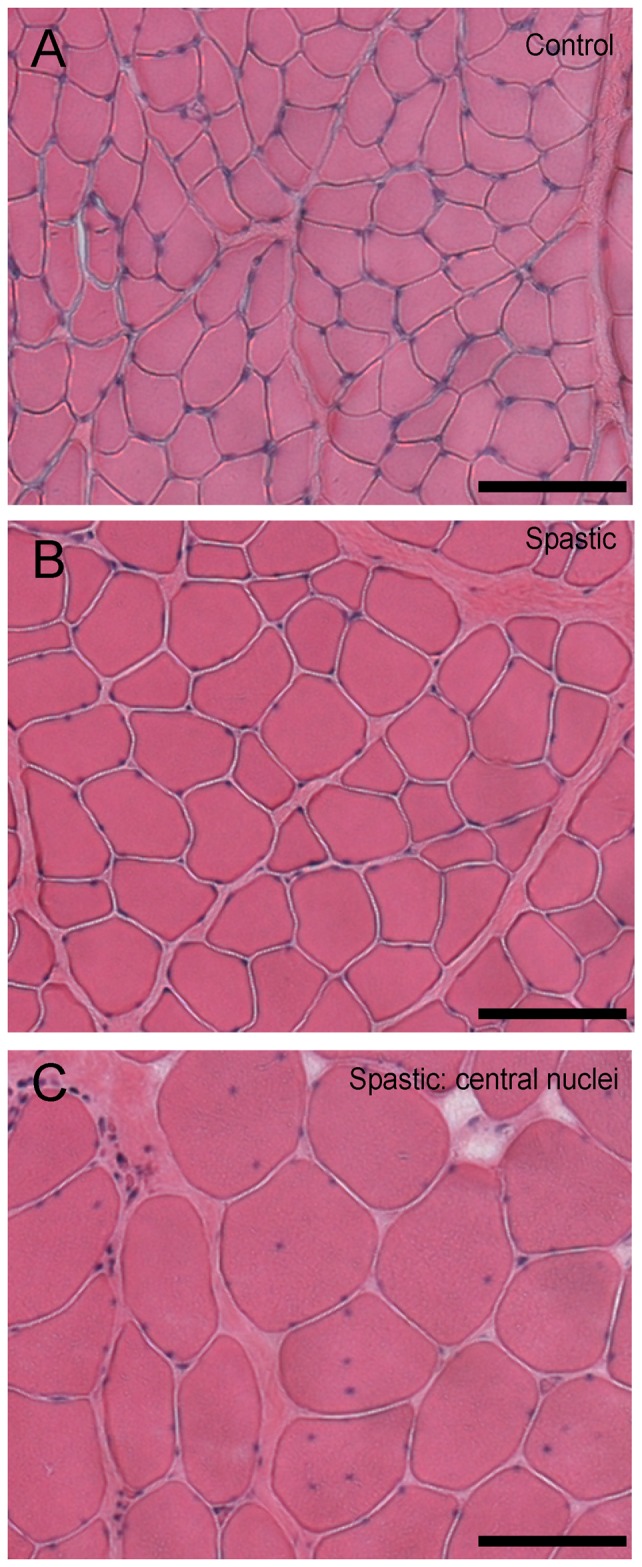
Light micrographic comparison of HE stained cross-sections of fascicles from spastic and control FCU. (**A**) Typical example of a cross-sectional image within control FCU. (**B**) Typical example of a cross-sectional image within spastic FCU. (**C**) example of the pathological sign of central nuclei observed in one CP patient exclusively. Bars represent 100 µm.

Normal localization of myonuclei was found for control subjects as well as patients (Fig. 3A and B), except for one patient. In that particular biopsy, central localization of nuclei was shown in a majority of myofibres (Fig. 3C). These results indicate that, in general, the spastic myofibres do not exhibit this sign of muscle damage.

#### Myofibre type distributions

**Figure 4:**
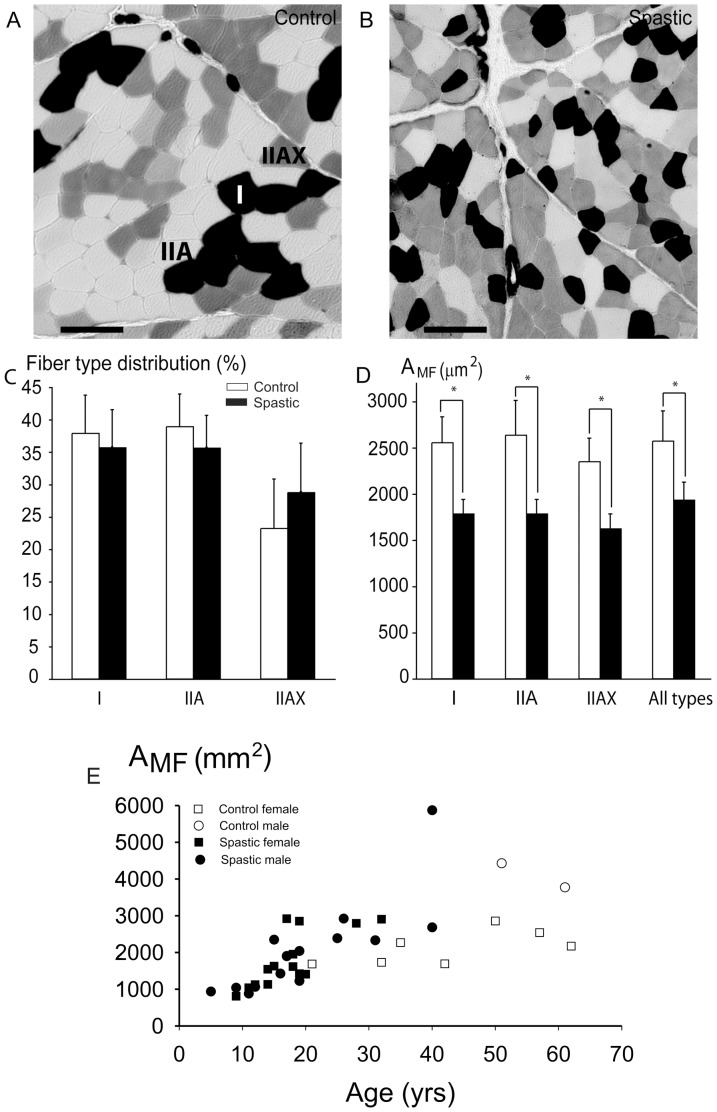
Myofibre typing and myofibre cross-sectional area within fascicles from spastic and control FCU. Typical examples of light micrographs of ATPase stained cross-section of biopsies from: (**A**) Control muscle, (**B**) Spastic muscle. Myofibre types I, IIA and IIAX are assigned; bars represent 100 µm. (**C**) Fibre type distribution within cross-sections from FCU. Fibre type distribution was not significantly different between CP (n = 26) and control (n = 10) samples. (**D**) For all fibre types, myofibre cross-sectional area (A_MF_) in all spastic samples was significantly smaller than in controls. (**E**). Individual data for A_MF_ plotted as a function of age. For spastic muscle of all ages taken together, Spearman's Rank coefficient of correlation for A_MF_ and age was significant and moderately high. Note that A_MF_ of spastic patients ≥20 years was not significantly different from that of (adult) control muscles. Means and SEM are shown; * indicates significant difference between spastic and control FCU.

Figure 4 shows examples of ATPase stained cross-sections of control and spastic fascicles. Counting of myofibres shows that in control subjects, the biopsies consisted on average for 38% of type I myofibres, 39% of type IIA and for 23% of type IIAX myofibres. The myofibre type distribution in spastic muscle (all CP children) did not differ significantly from that in control muscle (Fig. 4C). However, within the spastic group, the percentage type I fibres correlated negatively with age (Spearman's *ρ* = 0.46, *p*<0.016). In contrast, the percentages type IIA and IIAX did not correlate with age. In addition, fibre type distributions of spastic muscles, did not differ significantly between male and female CP patients.

#### Myofibre size

Regardless myofibe type, A_MF_ of spastic muscle (testing data for all CP subjects) was significantly smaller than that of control muscle (Fig. 4D, *p*<0.01). However, comparing A_MF_ exclusively for adult CP and control subjects (age ≥20 years), no significant difference was found (*t*-test). In addition, for spastic muscles, A_MF_ did not differ between adult male and female CP patients.

For spastic muscle of all ages taken together, Spearman's coefficient of correlation for A_MF_ and age was moderately high (*ρ* = 0.57, *p*<0.001). In combination with the lack of difference for A_MF_ between adult spastic and control muscles, this indicates that the correlation found for age and A_MF_, should be ascribed to age effects in young spastic myofibre size.

#### Intramuscular connective tissue

**Figure 5:**
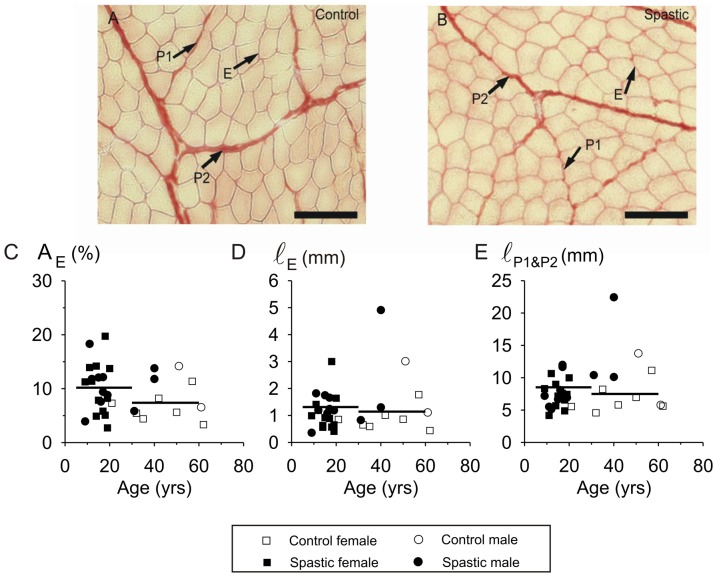
Variables of endomysium, primary and secondary perimysium in cross-sections of spastic muscle. Typical examples of light micrographs of Sirius Red stained cross-sections of FCU biopsies: (**A**) Sample of a 20-year old control subject. (**B**) Sample of an 18-year old CP subject. Bars represent 100 µm. (**C**) Individual data for cross-sectional area of endomysium within FCU plotted as a function of age. The mean (area) proportion taken up by endomysium (i.e. A_E_ expressed as % of total measured area) was not significantly different between cross-sections of CP (n = 23) and control subjects (n = 9). (**D**) Individual data for endomysium thickness (ℓ_E_) within FCU plotted as a function of age. The mean thickness of endomysium per myofibre cross-section was not significantly different in CP and control subjects. (**E**) Individual data for primary and secondary perimysial thickness (ℓ_P1&P2_) within FCU plotted as a function of age. Mean thickness of perimysium within cross-sections of spastic muscle were not different from those in control muscle. Horizontal lines indicate mean values.

**Figure 6:**
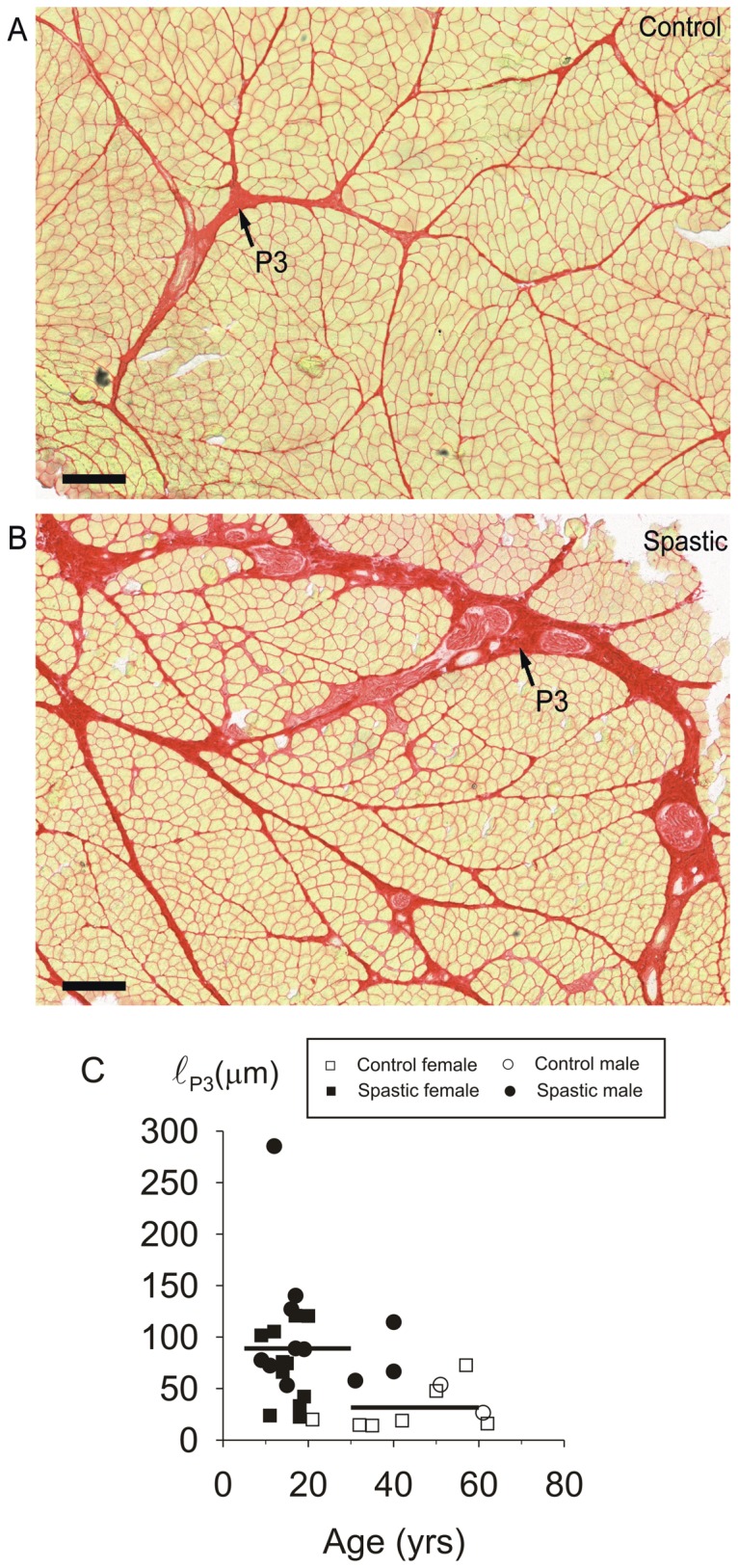
Increased thickness of tertiary perimysium within cross-sections of fascicles within spastic muscle. Typical examples of light micrographs of Sirius Red stained cross-sections of FCU biopsies: (**A**) control muscle. (**B**) Spastic muscle. Bars represent 250 µm. (**C**) Individual data of tertiary perimysium thickness within FCU biopsies plotted as function of age. Mean thickness of tertiary perimysium in FCU (indicated by horizontal lines) of spastic subjects (n = 23) was significantly higher than in FCU of control subjects (n = 9).

Figure 5 shows typical examples of Sirius Red stained cross-sections of control and spastic muscle biopsies. In general, we found no differences between spastic and control muscles for variables describing the endomysial or primary and secondary perimysial parts of intramuscular stroma: No significant differences were found for (1) A_E_ (Fig. 5C), (2) A_E_/MF (data not shown), (3) ℓ_E_ and (4) ℓ_P1&P2_ (Figs. 5 D-E). However, there was one exception: for the tertiary level of perimysium, a difference was found between control and spastic muscle (Fig. 6). The mean thickness of tertiary perimysium (ℓ_p3_) in spastic muscle (95.1±11.7 µm) was threefold that of control muscle (31.6±7.1 µm, *p*<0.01; Fig. 6C). However, note also that for some individual spastic muscles (n = 4 out of 23) the value of this variable (ℓ_p3_) was similar to that of controls, possibly indicating diverging properties of individual CP subjects. As the reverse is also true for some control subjects (i.e. fairly high values, n = 3), this indicates that individual variation of ℓ_p3_ may be a confounding factor.

For both groups, no significant rank correlations could be shown for age and any of these variables.

### Extramuscular connections and functional implications

In a majority of our CP subjects, thickening of intramuscular neurovascular tracts within spastic FCU may be indicative of adaptation to enhanced loading of these structures. Such loading likely occurs via extramuscular neurovascular tracts. To assess whether myofascial loading of spastic FCU occurs, we imaged effects of distal tenotomy on FCU during surgery (an example is shown, Movie S1). On distal FCU tenotomy the muscle retracts somewhat. Subsequent moving of the wrist joint back and forth between maximal dorsal and palmar flexion causes FCU to be lengthened and shortened repeatedly, despite the fact this muscle no longer crosses the wrist joint. These observations indicate the presence of myofascial loads exerted by neighbouring tissues onto FCU.

**Figure 7:**
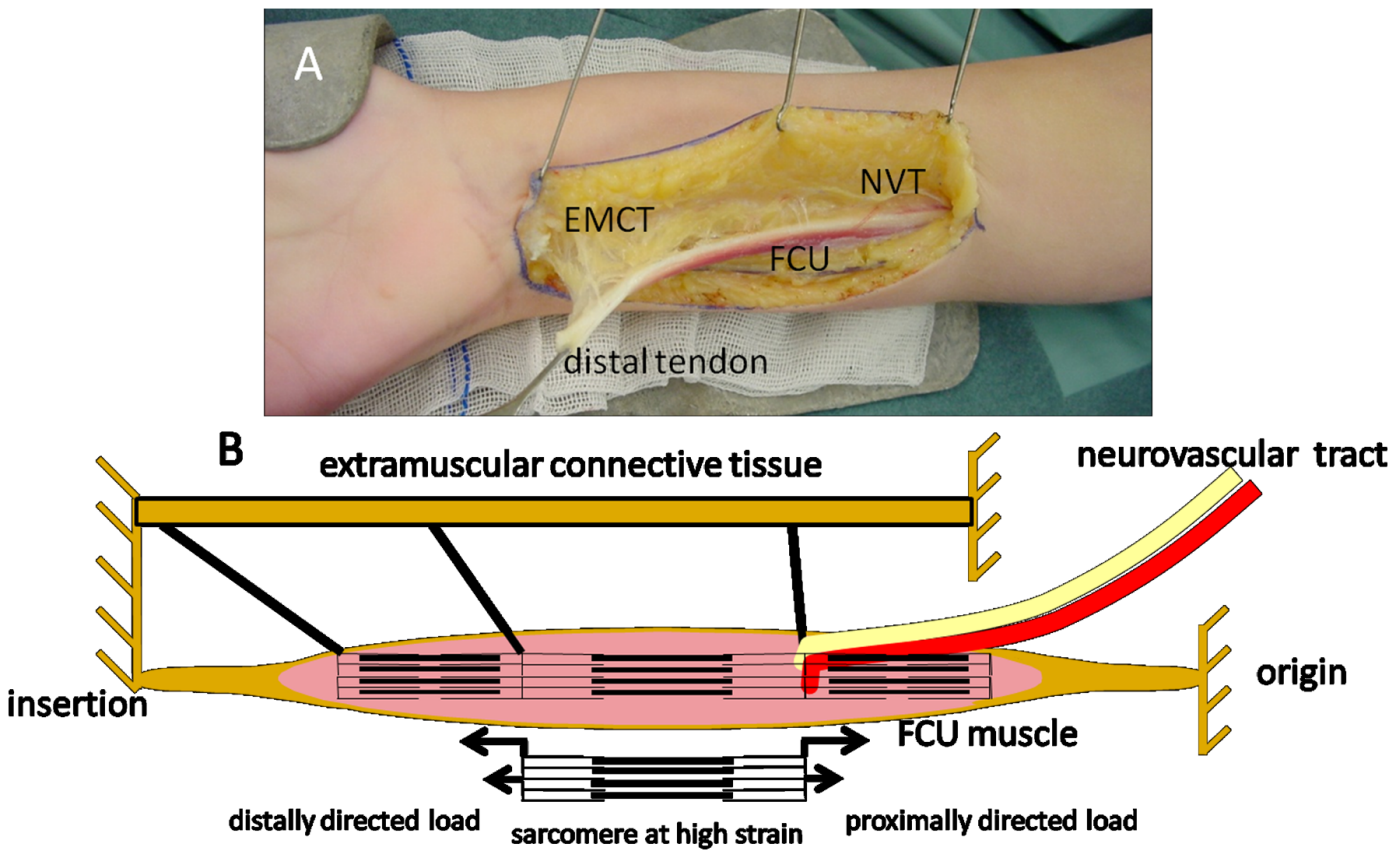
Neurovascular tract and simple schematics of connections. (**A**) Photograph of the tenotomised FCU and its myofascial connections to extramuscular connective tissue (EMCT) consisting of general fascia and neurovascular tracts (NVT). Note that, although not visible in the image, there are also connections to the epimysia of surrounding muscles (i.e. m. extensor carpi ulnaris and flexor digitorum superficialis/profundus). Via these connections, force can be transmitted between the stroma of FCU and extramuscular connective tissue such as general fascia, septa or NVT (i.e. epimuscular force transmission) and other muscles. This will cause loading in proximal as well as distal directions on a fraction of FCU. Distal loading of spastic FCU via myofascial connections has been shown after FCU tenotomy suggesting enhanced epimuscular loading (Movie S1) [64]. As branches of the NVTs generally enter the muscle from proximal directions, loading of the NVTs may chiefly yield proximally directed epimuscular loading of FCU. (**B**) Schematic diagram illustrating how concurrent proximal and distal epimuscular loads may cause high local sarcomere strains. Myofibres are represented by three sarcomeres in series, with myofascial connections to extramuscular connective tissues.

Tissues that remain intact after tenotomy consist of fascial extramuscular connective tissue surrounding the muscle belly. Important candidates for mediation of this effect are neurovascular tracts that enter the muscle belly (Fig. 7A) at several locations along its length.

## Discussion

The results of this study suggest that movement limitations in the spastic wrist are not explained by differences in FCU myofibre size, myofibre type or in thickness or quantity (absolute or normalized) of intramuscular connective tissues consisting of endomysia and perimysia. A notable exception is the tertiary perimysium being threefold as thick as spastic muscle compared to control muscle, suggesting accumulation of collagen in perimysium reinforcing major blood vessels, nerves and lymphatics,

### Limitations of the study

A major limiting factor of this study is the age difference between our patient and control groups. This age difference could not be prevented in our study design. Upper extremity tendon transfer surgery in CP patients often takes place in the second decade of life. But, due to medical ethical considerations, we were not allowed to include control subjects under the age of 18. However, we were able to include some CP patients aged over 20 years and used this subgroup for additional comparisons with the (adult) control group.

Our CP group consisted of similar numbers of males and females, but not our control group (n = 3 vs. n = 7, respectively). For the CP group, none of the variables studied differed between males and females. If this holds also for controls remains to be determined in future work.

Sarcomere length-tension measurements were performed on myofibre and fascicle segments rather than whole myofibres and fascicles. Consequently, we could not compare the series number of sarcomeres within whole myofibres with spastic and control muscle. However, passive and active length-force characteristics of partially dissected FCU in the spastic arm [38] were shown to be similar to those predicted for healthy muscle [39], [40]. This suggested that the overstretching of sarcomeres due to a decrease of number of sarcomeres in series may not be the primary cause for the movement limitation in the wirst joint [38].

Our measurements were performed at 20°C. However, sarcomere stiffness increases with temperature, particularly at high length [41]. At 37°C, effects of sarcomere length changes on myofibre and fascicle tension are expected to be higher than shown in our present results.

### Comparison of mechanical properties of fascicle and single myofibre segments

For myofibre segments obtained from spastic arm muscles, increased stiffness was reported previously [20], [23]. Also, based on previous reports [20], [23], the slope of the length-tension curve for spastic fascicle segments may be expected to be lower than for control segments [23]. However, in agreement with previous work from our group [38], neither differences in passive tension nor in slope of the sarcomere length-tension curve could be confirmed by our present results.

Comparison of tension of spastic myofibre segments as function of sarcomere length reported previously [20], [23] with our present results indicates that our values of stiffness of spastic myofibre and fascicle segments are higher than those reported previously. An explanation for this difference may be found in age differences: the mean ages of CP patients of the studies cited (7.8 [20] and 9.3 years [23]) were smaller than those of our present CP group (tested for mechanical properties, mean age 20.5 years). Although mechanical variables and age are not correlated (present results), it is conceivable that spastic myofibre and fascicle segments of CP children earlier in their childhood (i.e. age <10 years) may be more compliant than those of adolescents and adults. As muscle myofibre passive stiffness is associated with titin isoform expression [42], we speculate that during childhood a longer (more compliant) titin isoform may be replaced by a shorter (and stiffer) isoform. However, such young age effects do not explain the low stiffness of single myofibre segments reported for controls as the mean ages of that group were 37.4 [20] and 27.5 years [23]. This suggests that other factors are likely to be involved in explaining the differences results.

Comparison of our methods with those of previous studies shows methodological differences, which may contribute to the contrasting results: (1) some of the studies were conducted on several muscles from different muscle groups in the forearm, upper arm, and shoulder [20], [23] compared to only FCU biopsies in the present study. (2) Single myofibre and fascicle cross-sectional area analysis was previously based on the measurement of one diameter on the assumption of a circular shape [20], [23], yielding in either an over- or underestimation of cross-sectional area. Assuming a circular myofibre cross-section may lead to a mean deviation of 20% of the actual area, whereas our present assumption of an elliptical cross-section limits this error to a mean deviation of 4% of the actual area [43]. (3) An acknowledged drawback [6] of previous studies concerns tangent calculations of length-tension curves based on two points that were not equidistant at all times [20], [23]. In addition, sometimes points for tangent calculations were taken at sarcomere lengths up to 8.0 µm. This is likely to involve damage of the myofibre segment and/or fascicle segment [23]. In the present study we did not exceed 4.0 µm i.e. lengths which is far over the length of minimal thick and thin filament overlap (near 4.0 µm).

### Myofibre size

Regarding effects of spasticity on cross-sectional area of spastic myofibres, the literature remains inconsistent. Compared to control myofibres, some studies report growth deficits of spastic myofibres [12], [13], [15], [16], [17], whereas others report hypertrophy [13]. Also, similar myofibre sizes in spastic and control muscle have been reported [18], [44]. We found mean A_MF_ (data of all CP patients) to be significantly smaller in spastic muscle than in healthy muscle. In children, adolescents and young adults, A_MF_, mainly of leg muscles, increases with age until approximately 20 years of age (cf. [13], [33], [34], [36], [37], [45]). However, the fact that adult spastic and control muscles did not differ, indicates that there was no myofibre diameter growth deficit in adult spastic FCU. In any case, if there were a growth deficit within the CP group it disappears on reaching adulthood. Regarding myofibre growth of young CP children, our data does not allow us to exclude unequivocally such a growth deficit.

The coefficient of variance of A_MF_ has been claimed to be significantly higher in spastic muscle [17], [19]. Our results do not confirm that conclusion. It has been shown that oxidative capacity of myofibres is inversely related to myofibre cross-sectional area [46], [47]. Because no differences in myofibre type composition were found between spastic and control muscle, this variable does not likely affect our results regarding A_MF_.

For pennate muscle, such as FCU, myofibre diameter is a major co-determinant of the muscle slack and optimum length [10], [11] and as such the joint range of motion. Taken together, based on the above elaborations on myofibre size differences, we cannot attribute the occurrence of postural changes of the wrist in CP to changes in myofibre diameter.

### Myofibre typing

Skeletal muscle is well known to adapt to the quantity and type of neural activity [48]). Increase in muscular activity, for instance by means of exercise, may induce fast-to-slow transitions in myofibre types and expression of corresponding myosin isoforms [49]. Slow myofibre predominance has been reported in spastic muscle of the leg and arm [12], [13], [17], [18], however no comparison to a control group was made in those studies. Within the spastic forearm, flexor muscles are reported to have higher proportions of fast myofibres compared to extensor muscles [15], [19]. One of these authors later proposed that these results could best be explained by disuse [50]. In agreement with these results, the negative Spearman's Rank correlation found for myofibre type I percentage and age of our spastic FCU group indicates for this group that the fraction of type I fibres decreases with age. Therefore, spasticity may cause a myofibre type shift from slow to fast types, unless for controls similar effects would be present, in which case the effects should be ascribed to growth rather than as effects of spasticity. However, data on fibre type distributions in controls of similar age are required to draw any conclusions on this issue.

### Connective tissue

Without actual quantification, it has been suggested, that intramuscular connective tissue may be increased in spastic muscle [6], [12], [13], [21]. In addition, others reported that gene expression profiles of spastic muscle yields evidence for connective tissue remodelling [51] and the concentrations of connective tissue (not distinguishing endomysium, perimysium, and epimysium) in spastic vastus lateralis and semitendinosus muscles were reported to be increased [6], [21]. However, these studies made no distinction with regard to connective tissue structures within muscle. As the perimysium constitutes a relatively big fraction of intramuscular connective tissues [52] it is also considered a major contributor to extracellular sources of passive resistance to stretching of muscle [53], [54]. Hence, if contractures of spastic muscle would be caused by a change in intramuscular connective tissue content, the perimysium is likely an important factor in this. We distinguish the endomysium and three levels of perimysium.

In our biopsies, variables related to intramuscular connective tissue content (endomysium, as well as primary and secondary perimysium) did not differ between CP and control subjects, nor were they a function of age. Intramuscular connective tissue content in muscle of rodents much further along their life span than our subjects has been shown to be increased compared to that in young muscle [55], [56]. However, effects of age on intramuscular connective tissue content within human muscle is not unequivocal [57], [58], [59]). These studies indicate that fibrosis occurs within very old human muscle and even may be physiological.

The lack of increase in endomysium, as well as primary and secondary perimysium within spastic muscle is in accordance with our finding that the slopes of the length-tension curves of control and spastic single myofibre segments, as well as of control and spastic fascicles segments were not different. Therefore, our results for FCU are different from those for spastic vastus lateralis and semitendinosus muscles, in which collagen content, as assessed by hydroxyproline content, was increased [6]. This suggests that secondary effects of spasticity may differ between muscles in the upper and lower extremities.

Tertiary perimysial structures constitute a connection between collagen fibre reinforcements of intra- and extramuscular elements of neural, venous, arterial and lymphatic tissues. In fact the tertiary perimysia are continuations of (extramuscular) branches of the main neurovascular tracts. Note that the tertiary perimysia, that arethickened in spastic FCU, do not envelop fascicles or groups of fascicles from their origin to insertion, but rather enter and cross the muscle transversely at certain levels. By selection, tertiary perimysia were absent in the fascicle segments used for mechanical measurements. Enhanced thickness and presumably stiffness of such tertiary perimysium will more likely affect muscle function via its extramuscular connections by myofascial force transmission, rather than affect the stiffness of an isolated FCU. In other words its connections are crucial for enhanced stiffness.

Thickening and presumed stiffening of the tertiary perimysium as apparent in a majority of our CP subjects, suggests that, in spastic muscle, these structures are loaded relatively more than in controls. Such increased loading occurs by enhanced force transmission (further referred to as epimuscular force transmission) from the muscular stroma to structures other than the muscle's origin or insertion tendons [11]. Epimuscular force transmission may occur from the intramuscular stroma onto the epimysium of synergistic muscles or extramuscular neurovascular tracts, as well as onto other structures such as septa, general fascia, interosseal membrane and periost. Epimuscular loads exerted on a muscle can have distal or proximal directions [60], [61], [62]. In CP patients, the presence of enhanced distal loads on FCU seems evident from the observations that after distal FCU tenotomy the muscle is kept at length and that subsequently extending the wrist stretches both passive (Movie S1) and active FCU muscle [63], [64]. These distal loads applied to FCU are exerted via extramuscular connective tissue structures (Fig. 7). Branches of the neurovascular tracts that are embedded in these structures generally enter the muscle from proximal directions. If neurovascular tracts are thicker, such loading will chiefly yield in proximal epimuscular loads on FCU. Myofascial force transmission via such tracts has also been shown to be effective in rodents [61]. If the extramuscular connective tissue is stiffer in spastic patients, extending the wrist will cause simultaneous proximally and distally directed epimuscular loads to be exerted on FCU.

A very special effect of oppositely directed myofascial loads on FCU is that force can be transmitted locally through the muscle without being exerted at its origin and insertion [22]. Because of this condition, it is feasible that a very small fraction of the sarcomeres arranged in series within FCU myofibres is kept at high length, whereas simultaneously the remainder of the sarcomeres within those fibres are at low lengths (Fig. 7) [22]. Note that in spastic patients, it is conceivable that such specific local conditions have sizable effects on joints involved without being very apparent in muscular morphology.

The following conclusions are drawn. No significant differences between control and spastic muscle were found regarding slope of the passive length-tension curves of myofibre segments, cross-section or myofibre type proportions. The altered connective tissue composition of FCU, secondary to spasticity, is manifest exclusively by thickening of its tertiary perimysium in a majority of our CP subjects. This is in contrast to assumptions that spasticity may cause thickening of all of the muscular connective tissue stroma. There are indications that tertiary perimysial tracts in mechanical interaction with extramuscular connective tissues surrounding FCU may play a role in the aetiology of the typical CP wrist joint postures. The substantial individual variation, however, also indicates that this may not be the exclusive mechanism contributing to these wrist postures.

## Supporting Information

Movie S1 **Passive excursion of the FCU of a child with cerebral palsy after tenotomy of the distal tendon.** The movie shows a cut distal tendon of FCU in a patient undergoing a tendon transfer surgery. Note that, after distal tenotomy, the muscle is prevented from shortening. As the wrist is moved alternately into full extension and flexion, FCU lengthens and shortens, respectively. The distally directed loads exerted on FCU have to be transmitted via extramuscular connective tissue (i.e. shared epimysia of neighbouring muscles, and/or general fascia and septa), whereas the proximally oriented loads are likely to be exerted onto FCU via neurovascular tracts.
(MPG)
